# Factors associated with *Toxoplasma gondii* infection in confined farrow-to-finish pig herds in western France: an exploratory study in 60 herds

**DOI:** 10.1186/s13071-016-1753-5

**Published:** 2016-08-24

**Authors:** V. Djokic, C. Fablet, R. Blaga, N. Rose, C. Perret, O. Djurkovic-Djakovic, P. Boireau, B. Durand

**Affiliations:** 1 Ecole Nationale Vétérinaire d’Alfort, ANSES, INRA, Université Paris-Est, Laboratoire de santé animale de Maisons-Alfort UMR BIPAR, Maisons-Alfort, France; 2 ANSES, Unité Epidémiologie et Bien-Etre du Porc, Université Européenne de Bretagne, Laboratoire de Ploufragan-Plouzané, Ploufragan, France; 3 ANSES, Ecole Nationale Vétérinaire d’Alfort, INRA, Université Paris-Est, Laboratoire de santé animale de Maisons-Alfort, UMR BIPAR, Maisons-Alfort, France; 4 Institute for Medical Research, National Reference Laboratory for Toxoplasmosis, University of Belgrade, Dr. Subotića 4, P.O. Box 102, Belgrade, 11129 Serbia; 5 ANSES, Université Paris-Est, Laboratoire de santé animale de Maisons-Alfort, Epidemiology unit, Maisons-Alfort, France

**Keywords:** *Toxoplasma gondii*, Intensive pig farm, Risk factors

## Abstract

**Background:**

Infection by *Toxoplasma gondii* postnatally can occur after ingestion of contaminated meat or water (tissue cysts/oocysts). In Europe, percentage of meat borne infections is estimated between 30 and 63 %, out of which pork makes the most important source. The aim of this study was to (i) investigate the seroprevalence of *T. gondii* in intensive pig farms from western France; and (ii) identify the risk factors associated with seropositivity.

**Methods:**

Data were collected between November 2006 and February 2008 in 60 intensive farrow-to-finish farms, where sera were taken from 3595 fattening pigs, weaned and suckling piglets. Information about three classes of potential seropositivity risk factors were obtained through a questionnaire concerning: (i) breeding characteristics; (ii) farm management; and (iii) husbandry and hygiene. The modified agglutination test (MAT) was used for detection of specific anti *T. gondii* antibodies in pig sera, starting from 1/6 dilution.

**Results:**

The overall proportion of seropositive animals was 6.9 %, but the proportion of herds with at least one positive pig was 100 %. Multivariate logistic mixed model showed an increased seropositivity risk in weaned compared to suckling piglets, and a decreasing risk for mid-sized and large farms. The presence of a Danish entry facility, that clearly separates clean and dirty areas, had a protective effect on *T. gondii* seropositivity as well.

**Conclusions:**

The observed proportion of herds with at least one *T. gondii* seropositive animal provides further evidence that even in confined conditions of pig breeding, infection occurs, and is common. The highest risk for acquiring *T. gondii* is at the end of weaning period. Smaller confined pig farms demonstrate higher *T. gondii* seropositivity levels. This study also showed that Danish entry on farm buildings provides effective protection against *T. gondii*.

**Electronic supplementary material:**

The online version of this article (doi:10.1186/s13071-016-1753-5) contains supplementary material, which is available to authorized users.

## Background

*Toxoplasma gondii* is one of the world’s most widespread food borne pathogens. The parasite from phylum Apicomplexa is adapted for infection of all warm-blooded animals including humans. The characteristic of *T. gondii* is that parasites can be transmitted directly among intermediate hosts, including pigs, by predation or scavenging without need for the sexual part of the life-cycle in felids. The ingestion of food or water contaminated with different parasitic life stages (oocysts/tissue cysts) are the two major routes for postnatal transmission of *T. gondii* [1].

Infection in humans is generally benign, characterized by mild, flu-like symptoms. However, if maternal infection occurs in pregnancy, *T. gondii* may cause serious damage on the developing foetus; and in immunosuppressed individuals lesions of nervous system and eyes can occur with even life-threatening consequences [2]. Due to its zoonotic character, this parasitosis represents an important public health hazard.

The percentage of meat-borne infections of the total number of toxoplasmosis patients has been estimated between 30 and 63 % in Europe [3]. Along with sheep, pigs are the species mostly associated with human *T. gondii* infection [4]. Nowadays three pig farming systems are recognized: (i) intensive, (ii) free-range (outdoor) and (iii) organic farming. In intensive farms animals are kept in strictly confined conditions, without any access to outside environment. Pigs are separated in categories, defined as batches (contemporary group of pigs), from where all animals are transported in the same time (all-in, all-out). In contrast, both organic and outdoor pigs have access to natural light, weather conditions and contact with other animal species. Mainly, organic pigs are bred in large open space pens without closed facilities, fed by seasonal food without chemical processing, and the system all-in, all-out is not practiced.

Various levels of seroprevalence have been reported during the last decade in European pigs and wild boars. In five European countries (the Netherlands, Sweden, France, Latvia and Switzerland), published estimates allow comparing seroprevalence levels observed in intensively bred pigs (the dominant breeding system in Europe), in organic/outdoor pigs and in wild boars. A common trend is observed in four of these five countries, with low seroprevalence levels in intensively bred pigs (0.4 % in the Netherlands [5], 1 % in Sweden [6], 2.9 % in France [7], and 0.4 % in Latvia [8]), medium seroprevalence levels in organic/outdoor pigs (5.6 % in the Netherlands [5], 8 % in Sweden [9], 6.3 % in France [7], and 6.2 % in Latvia [8]), and high seroprevalence levels observed in wild boars (24 % in the Netherlands [10], 50 % in Sweden [9], 23 % in France [11], and 33 % in Latvia [8]). An opposite trend had been reported in Switzerland [12], with similar seroprevalence observed in intensively bred (14 %) and organic/outdoor pigs (13 %), and a lower seroprevalence observed in wild boars (7 %). The similar trends and seroprevalence levels observed in the four above countries are coherent with the hypothesis that wild boar and organically/free-ranged pigs get infected mainly from an oocyst-contaminated environment by rooting, or by consumption of infected rodents, whereas pigs not exposed to oocyst-contaminated environment, nor infected rodents (i.e. pigs from confined, intensive farms) appear to be less often seropositive.

Routine *T. gondii* testing of pigs, destined for human consumption has not been introduced in any country [13]. Therefore the existence and location of endemic areas, the sources of pig infection, the frequency of infected pigs on the markets, and the role these animals play in human *T. gondii* epidemiology remain unknown.

In 2013, a French nation-wide study of *T. gondii* in intensively bred pigs slaughtered for human consumption was conducted. Samples from randomly selected piglets and fattening pigs were taken in 26 slaughterhouses. The resulting seroprevalence rates were 2.6 % in piglets and 2.9 % in fattening pigs [7]. However, the study design (based on samples taken in slaughterhouses) did not allow estimating the proportion of infected farms, or the proportion of seropositive animals in these farms.

Therefore, the aim of the present work was to analyse the presence of *T. gondii* in intensive pig farms and to investigate: (i) the farm-level and within farm proportion of seropositives, and (ii) factors associated with seropositivity. For this purpose, sera and data related to herd characteristics, biosecurity, management and housing conditions (obtained by questionnaire) were collected from 60 confined farrow-to-finish farms. All studied farms were located in western France, the region were almost 60 % of total country’s pork production is located [14, 15].

## Methods

### Database and serum bank

Sera samples and epidemiological data were obtained from a previous study, conducted from November 2006 to February 2008 in 125 farrow-to-finish pig productions from western France with more than 100 sows per farm [14]. In each of these 125 farms, blood samples were collected from pigs. A face-to-face interview, which included 610 questions, was conducted with the farmer, as well as herd investigations (using a standardized protocol with 257 measurements and observations, see Additional file 1 for the part of the questionnaire used for the present study), by the Swine epidemiology and welfare unit (ANSES), as described in [14]. The resulting dataset included a general description of the herd and of its neighbourhood, and data about biosecurity and hygiene practices, as well as management and animal husbandry. The serum bank included a total number of 7500 sera from pigs of 4 age classes: <1 month, 1–2 months, 2–3 months and >3 months.

### Herd selection and risk factors

A written consent from farmers (represented by their organisations of producers) was asked for the use of pig sera and data in the present study. A farm was considered as *T. gondii*-positive if specific antibodies were found in at least one pig. The *a priori* estimate of the proportion of positive farms was set at 20 %. For a relative precision of 5 %, the sample size was calculated to be 60 farms, which were randomly chosen among 125 available from the database. Assuming a within-herd prevalence of 5 %, the number of samples per farm necessary to detect *T. gondii* presence (i.e. to obtain at least one positive serum) at a 95 % confidence level was 60 sera. These 60 sera were equally distributed in the 4 age classes. Thus, for each of the 60 chosen farms, 60 pig sera were randomly selected in the serum bank: 15 sera from <1 month suckling piglets, 15 sera from 1 to 2 months weaned piglets, 15 sera from 2 to 3 months fattening pigs, and 15 sera from >3 months fattening pigs.

On all farms pigs were kept in completely confined conditions, without any outside access neither for piglets or sows. Farm buildings were separated according to each of the 4 age classes: farrowing rooms for suckling piglets (and sows) from birth to one month of age, weaning rooms for one to two months old piglets, fattening rooms for animals of 2–3 months of age, and rooms for >3 months old animals.

The dataset associated to farm- and age-class specific serological results (number of tested and number of positive animals) included both room-level and farm-level variables. Room-level variables were the type of floor (slatted floor, straw, combination of slated and concrete floor) and the feed distribution method (automatic feeders or manual). Farm-level variables included hygiene practices: the use of specialized boots (yes/no) and clothes (yes/no), the compliance with all-in, all-out management (time in days during which pens/rooms/buildings were left empty in post-weaning and post-fattening processes), and the presence of a Danish entry facility (yes/no): a closed facility with at least two doors that open successively, upon entering the breeding rooms. All farms had controlled airflow, used pressurized steam for cleaning and specialized external company always performed rodent control; these elements were thus not considered in the analysis. Farm-level variables also included the number of sows (on a logarithmic scale), used as a proxy for herd size. Finally the presence of dairy cows (yes/no), beef cattle (yes/no), as well as sheep (yes/no), goats (yes/no) and poultry (yes/no) breeding facilities on the same farm were considered.

A total number of 14 individual-level (1 variable), room-level (2 variables) and farm-level (11 variables) variables were thus considered (Table 1).

**Table 1:** Farm-level, room-level and individual-level variables considered as *T. gondii* seropositivity risk factors in pigs from confined farrow-to-finish farms. Out of risk factors for which exposure is not rare (>5 farms), presence of Danish entry facility, farm size, dairy production, feed distribution mechanism, and age of animals have a statistically significant influence on *T. gondii* seroprevalence in pigs. These factors entered logistic mixed regression model

Risk factor	*n*	Animals - positive/total (%)	*P*-value
Farm level	60 farms		
Use of specialized boots			0.94
No	20	95/1198 (7.9 %)	
Yes	40	156/2397 (6.5 %)	
Use of specialized clothes			0.18
No	20	92/1193 (7.7 %)	
Yes	40	159/2402 (6.6 %)	
Empty period (days) of farrowing rooms			0.89
Empty period (days) of rooms for >3 months old animals			0.30
Danish entry			<0.001
No	43	172/2487 (6.9 %)	
Yes	17	77/1048 (7.3 %)	
Number of sows per farm			<0.001
Presence of dairy cattle			<0.001
No	54	207/3236 (6.4 %)	
Yes	6	44/359 (12.3 %)	
Presence of beef cattle			0.03^a^
No	56	242/3356 (7.2 %)	
Yes	4	9/239 (3.8 %)	
Presence of sheep			0.005^a^
No	58	234/3475 (6.7 %)	
Yes	2	17/120 (14.2 %)	
Presence of goats			0.21^a^
No	59	249/3535 (7.0 %)	
Yes	1	2/60 (3.3 %)	
Presence of poultry			0.01^a^
No	57	246/3415 (7.2 %)	
Yes	3	5/180 (2.8 %)	
Room level	234 rooms		
Floor type			0.97
Slatted floor	225	242/3457 (7.0 %)	
Straw	1	1/15 (6.7 %)	
Slatted and concrete floor	8	8/123 (6.5 %)	
Feed distribution			0.09
Automatic system	79	73/1219 (5.9 %)	
Manual distribution	155	178/2376 (7.5 %)	
Individual level	3595 pigs		
Age			0.002
<1 month	545	29/545 (5.3 %)	
1–2 months	438	36/438 (8.2 %)	
2–3 months	790	76/790 (9.6 %)	
>3 months	1822	110/1822 (6.0 %)	

^a^Variables with a low exposure level (less than 5 farms or rooms) were excluded from the multivariate analysis

### Serology testing

Detection of *T. gondii* IgG antibodies was performed on sera by MAT, as described by Halos et al. [16], since this is a species-independent serological test. MAT is considered as a gold standard for the detection of *T. gondii* antibodies in animals and meat [17–20]. The National Reference Centre for toxoplasmosis in Reims, France, provided the antigen. Four two-fold dilutions were made starting from 1/6 to 1/50. All results were assessed qualitatively (negative/positive) regardless of dilution.

### Statistical analysis

The overall animal- as well as age class-specific proportions of seropositive animals was calculated, with the associated exact binomial confidence intervals (95 % CI). Because of the relatively small size of the farm sample (60 farms), four risk factors for which exposure was rare (less than 5 rooms or farms) were excluded from the analysis (Table 1). The univariate association between the serological status of pigs (positive/negative) and the independent variables (i.e. the age class and the nine room- and farm-level variables included in the analysis) was analysed using logistic regression models and likelihood ratio tests. Variables for which *P*-values were ≤0.20 were selected for inclusion in the multivariate model. Verifying that variance inflation factor was <5 for each of the selected variables checked absence of significant multi-collinearity. A logistic mixed regression model was then used, in which the dependent variable was the pig *T. gondii* serological status (positive/negative). The selected explicative variables were included as fixed effects, and the farm was included as a random effect. Significance threshold was set to 0.05.

All statistical analyses were performed using R [21] (http://www.R-project.org/), using the *MASS*, *lme4* and *car* packages [22–24].

## Results

### Animal, farm and within-farm levels of *T. gondii* frequency of seropositivity

A total number of 3600 sera (60 farms and 60 sera per farm) were taken from the serum bank for serological analysis. Five of these sera gave inconclusive MAT results; hence the analysis was eventually performed on 3595 individual serological results. All herds had at least one seropositive pig, which brought the frequency of seropositive farms at 100 % (60/60) (95 % CI: 94–100 %). When only one age category was considered, the proportion of seropositive farms (i.e. farms with at least one seropositive animal in the considered category) was 50 % (18/36) for suckling piglets (<1 month) (95 % CI: 34–66 %), 48 % (29/60) for weaned animals (1–2 months old) (95 % CI: 35–61 %), as well as for 2–3 months old fattening pigs (20/42) (95 % CI: 33–63 %). The proportion of farms with at least one seropositive pig older than 3 months was 82 % (49/60) (95 % CI: 72–92 %).

The overall proportion of seropositive animals was 7.0 % (251/3595; 95 % CI: 6.2–7.9 %). It varied according to the age class: 5.3 % in <1 month old suckling piglets (29/545; 95 % CI: 3.6–7.6 %), 8.2 % in 1–2 months of age weaned animals (36/438; 95 % CI: 5.8–11.1 %), 9.6 % in pigs 2–3 months old (76/790; 95 % CI: 7.7–11.9 %), and 6.0 % in animals older than 3 months (110/1822; 95 % CI: 5.0–7.2 %) (Table 1).

The within-herd proportion of seropositive animals ranged between 2 and 28 %. This range varied according to the age class: 0–27 % in <1 month old suckling piglets, 0–40 % in 1–2 months of age weaned animals, 0–93 % in pigs 2–3 months old, and 0–30 % in animals older than 3 months.

### Risk factor analysis

Based on univariate analyses, six variables were selected for inclusion in the multivariate model: the presence of dairy cattle, the herd size (number of sows on a logarithmic scale), the use of specialised clothes inside production units, the feed distribution method, the presence of a Danish entry facility, and the age class (Table 1).

In the multivariate logistic mixed model (Table 2), a significant association between age class and *T. gondii* seropositivity risk was observed, with increasing odds-ratios (OR) for 1–2 months old weaned piglets (OR = 1.9, *P* = 0.04) and for 2–3 months old fattening pigs (OR = 2.0, *P* = 0.002) when compared to suckling animals (<1 month of age). However, in >3 months old pigs, the seropositivity risk was not significantly different from the one observed in <1 month piglets (*P* = 0.29) (Table 2). The number of sows (a quantitative variable, on a logarithmic scale, used as a proxy for herd size) had a significant effect on pig seropositivity risk, with an OR of 0.87 (*P* = 0.04) for an increase of herd size from the 25 % percentile of its distribution in the sample (139 sows/farm) to the 75 % percentile (251 sows/farm). No significant association was observed between seropositivity risk and the presence of dairy cattle (*P* = 0.24), the feed distribution method (*P* = 0.38), and the use of specialized clothes inside the production units (*P* = 0.77). The presence of a Danish entry facility had a protective effect on pig seropositivity risk with an OR of 0.58 (*P* = 0.04) (Table 2).

**Table 2:** Logistic mixed regression model of *T. gondii* infection intensity in pigs from farrow-to-finish farms

Risk factor	Value	Odds-ratio (95 % CI)	*P*-value
Age class	<1 month – suckling piglets	1	
	1–2 months – weaned piglets	1.9 (1.0–3.2)	0.04
	2–3 months – young fattening pigs	2.0 (1.3–3.2)	0.002
	>3 months – fattening pigs	ns	0.29
Herd size (number of sows on a logarithmic scale)	Increase from the 25 % (139 sows) to the 75 % percentile (251 sows) of the observed distribution	0.87 (0.8–0.9)	0.04
Presence of dairy cattle	No	1	
	Yes	ns	0.24
Presence of a Danish entry facility	No	1	
	Yes	0.58 (0.3–0.9)	0.04
Use of specialised clothes inside production units	No	1	
	Yes	ns	0.77
Feed distribution method	Automatic feeder	1	
	Manual distribution	ns	0.38

This model demonstrates an increased seropositivity risk in weaned piglets (1–2 months of age), and 2–3 months old animals as compared to suckling piglets. Seropositivity risk is lower in pigs from larger farms than in animals from small producers. Use of Danish entries on the farm decreases seropositivity risk as well. Presence of dairy production, the use of specialised clothes per room, and methods of feed distribution did not influence *T. gondii* seropositivity of pig bred in confined farms

## Discussion

For the last 15 years, surveys of *T. gondii* in pigs raised and consumed throughout Europe were reported from 14 countries, in which various age categories were analysed (piglets, fattening pigs, reproduction animals). In 11 studies sample collection was done at the slaughterhouse, allowing estimation of animal- and national-level prevalence, but not of farm-level and within-farm prevalence. Farm-level studies showed that the major risk factors associated with *T. gondii* infection in pigs were the breeding method, the animal age, the presence of cats, and the absence of rodent control [5, 7, 8, 17, 25–29]. Out of five studies that reported different prevalence between intensive and outdoor farming, four showed significant differences between these two housing types, and only Berger-Schoch et al. [12] reported contradictory results in Switzerland. Studies from Portugal, Spain, France, Serbia, Switzerland, Slovakia, and Germany showed that older age categories, from any housing method (intensive and outdoor), showed higher seroprevalence levels [7, 12, 26, 29–32]. As a source of contamination for intensively bred pigs in Europe, studies from Spain and Romania identified cats, and reports from Italy and Spain associated lack of rodent control [26, 27, 33, 34]. At the same time, a study from the Netherlands showed significantly lower *T. gondii* seroprevalence in intensive - strictly confined pig farms, without access of cats and rodents into buildings [5]. The same risk factors for *T. gondii* infection of pigs, age of animals, presence of cats, absence of rodent control, type of farm management, were reported in studies from Brazil and Mexico [35–38]. A study from Paraiba state in Brazil found that feeding with leftovers increases chances for pig infection with *T. gondii* as well [35]. In the Brazilian state of Toledo, the risk for parasites infection was higher on farms where workers were not assigned to specific area of the farm, other animals had access to pig feeders and drinkers, and where mean piglet number was low [39]. For USA indoor-raised pigs rodent control measures and carcass disposal methods were identified as main risk factors for *T. gondii* seropositivity [40]. In central China, except for the presence of cats and density of pig population in the pens, the presence of mosquitos and flies, as well as semi-patency pens and low frequency of scavenging represent risk factors associated with *T. gondii* seropositivity [41].

In the present study, the piglets and fattening pigs from closed farm facilities in France were tested for the presence of anti-*T. gondii* antibodies. For this purpose, pig sera collected from 2006 to 2008, for respiratory diseases study in farrow-to-finish farms from western France were used. Samples have been kept in sera bank at −80 °C without defrosting for the last ten years, which made them valuable for the present analysis, as in previous studies (e.g. [42]). At the same time, for the last ten years biosecurity and hygiene systems in farrow-to-finish farms changed insignificantly, thus the results of this analysis can be applied to nowadays pig producers, as well. This is the first in depth analysis of individual and on farm factors that allows identifying the most vulnerable moments in pig breeding for *T. gondii* infection, as well as conditions that favour parasite spread throughout the buildings of farrow-to-finish farms.

The proportion of 100 % positive farms indicates a widespread contamination of pig farms. Animal-level seropositivity rate was relatively high (7 %) and within-herd seropositivity rate reached 93 % in pigs 2–3 months old of one of the 60 farms. The animal-level seropositivity rate of 7.0 % reported here is similar to the seroprevalence reported in Serbia, Portugal and Austria, in pigs from same type of confinement [31, 32, 43]. However, the samples analysed in the present study were taken from a previous cross-sectional survey, in which inclusion criteria was the history of respiratory diseases [14], and therefore cannot be considered representative of the French farrow-to-finish farms, although no obvious association exists between the occurrence of respiratory diseases and *T. gondii* (other than hygienic conditions which were explicitly considered in the present study).

The multivariate model of pig seropositivity indicated a significant association between seropositivity risk and age class. Duration of maternal antibodies from naturally infected sows in piglets lasts from three to six weeks of life [44] and in experimental conditions up to 17 weeks [45]. In this period, with the decrease in the concentration of specific *T. gondii* antibodies, young animals become susceptible to infection. The earliest age piglets can get infected orally in experimental conditions is between the fourth and fifth week of life [46]. This goes to show that in piglets younger than one month, a *T. gondii* seropositive result may be attributed to maternal antibodies. Furthermore, in experimentally infected piglets, newly formed antibodies can be detected only after 2–3 weeks [46, 47]. Therefore, in the 1–2 months old animals, significantly higher seropositivity risk than in suckling piglets suggests that the seropositive results may in part be due to newly established infections. Furthermore, the higher odds-ratio observed for 2–3 months old animals implies that most probably infection occurs at the end of second month of life, when feeding becomes more diverse. In the cross-sectional survey of respiratory diseases upon which the present study is based [14] some pig sera were analysed for infectious agents responsible for alterations of pig’s immune system, such as Porcine circovirus (PCV) and Porcine reproductive and respiratory virus (PRRSV). These analyses aimed at documenting viral circulation at the batch level, and the individual serological results were unfortunately not available for the 3595 pig samples used for *T. gondii* analysis. For this reason, seropositivity for PCV and/or PRRSV could not be considered as risk factors of *T. gondii* seropositivity risk.

Only the study from Serbia showed farm size as a risk factor [31]. Our study shows, as well, that for pigs risk of acquiring *T. gondii* infection was higher in animals living in small pig farms, which may suggest a lower technical and hygienic level in small producers.

The present study shows that installation of a Danish entry facility in the pig breeding farms decreases the *T. gondii* seropositivity risk. In the confined conditions of intensive pig farms, Danish entry is a closed facility with several doors that open successively, one at a time, upon entering and exiting the breeding facility. In the majority of cases it serves for boots and clothes change and sanitation, and prevents direct entering into the buildings. In these conditions even if there are other animal species (potential sources of *T. gondii* infection such as rodents, small birds and cats), their contact with pigs is less likely.

## Conclusion

The described *T. gondii* seropositivity in intensively bred pigs from western France, complements the results of a previous animal-level, nation-wide study, by demonstrating that even in confined conditions of pig breeding, parasite infection exists and that current biosecurity and hygiene measures have not met satisfactory standards for production of Toxoplasma free pork. This is the first in depth analysis of intensive farm conditions and their influence on seropositivity risk. Current study shows that maternal *T. gondii* antibodies are present in suckling piglets, and suggests that the risk of acquiring parasite is the greatest at the end of weaning period. The presence of other livestock productions on the same farm does not appear to have any impact on *T. gondii* infection. However, small farms where pig breeding is probably not the primary activity, demonstrated increased *T. gondii* levels of seropositivity risk, despite the confined conditions. This study also shows that Danish entry facilities on farm buildings appear to be an effective protection measure from *T. gondii* infection. Additional studies are necessary to focus on specific reservoirs of infection in intensive farms.

## Additional file

Additional file 1: Questionnaire used to collect epidemiological data. (DOCX 23 kb)

## Abbreviations

CI: Confidence intervals
MAT: Modified agglutination test
OR: Odds-ratios
PCV: Porcine circo virus
PRRSV: Porcine reproductive and respiratory virus
