# Supplementary material for: Factors associated with Toxoplasma gondii infection in confined farrow-to-finish pig herds in western France: an exploratory study in 60 herds
Source: Parasit Vectors. 2016 Aug 24;9(1):466. doi: 10.1186/s13071-016-1753-5 (PMC4997743; doi:10.1186/s13071-016-1753-5)
Supplement: Additional file 1: — Questionnaire used to collect epidemiological data. (DOCX 23 kb) [file 13071_2016_1753_MOESM1_ESM.docx]

| Farm Number |
| --- |
| …………………………. |
| Region? |
| …………………………. |
| Commune**?** |
| …………………………. |
| Farm description |
| Type of swine herd? |
| - Farrow to finish |
| - Post-weaning and finishing |
| - Finishing |
| Dairy production on the farm? |
| - No |
| - Yes |
| Beef cattle on farm? |
| - No |
| - Yes |
| Horses on farm? |
| - No |
| - Yes |
| Sheep production on the farm? |
| - No |
| - Yes |
| Goat production on the farm? |
| - No |
| - Yes |
| Poultry production on the farm? |
| - No |
| - Yes |

| Swine herd |
| --- |
| Number of sows in the swine breeding herd? |
| …………………………. |
| Number of piglets in the farrowing section? |
| …………………………. |
| Number of nursery pigs on the farm? |
| …………………………. |
| Number of fattening pigs? |
| …………………………. |
| Does the staff wear specific clothes in the swine facilities? |
| - No |
| - Yes |
| Does the staff wear specific boots in the swine facilities? |
| - No |
| - Yes |
| Duration of the down period (after cleaning-disinfection and before restocking) in the fattening section (number of days)? |
| …………………………. |
| Duration of the down period (after cleaning-disinfection and before restocking) in the nursery (number of days)? |
| …………………………. |
| Washing procedure when cleaning the rooms? |
| - High pressure |
| - Low pressure |
| Floor type |
| - Fully slatted floor |
| - Partly slatted floor |
| - Deep-litter |
| Feeding type |
| - Wet feeding |
| - Dry feeding (pellets) |
